# Social Inequalities in Non-ischemic Cardiomyopathies

**DOI:** 10.3389/fcvm.2022.831918

**Published:** 2022-03-07

**Authors:** Eisuke Amiya

**Affiliations:** ^1^Department of Cardiovascular Medicine, Graduate School of Medicine, University of Tokyo, Tokyo, Japan; ^2^Department of Therapeutic Strategy for Heart Failure, University of Tokyo, Tokyo, Japan

**Keywords:** cardiomyopathy, inequality, race, sex, hypertrophic cardiomyopathy, dilated cardiomyopathy

## Abstract

Heart failure (HF) has various characteristics, such as etiology, clinical course, and clinical characteristics. Several studies reported the clinical findings of the characteristics of non-ischemic cardiomyopathy. There have been issues with genetic, biochemical, or pathophysiological problems. Some studies have been conducted on non-ischemic cardiomyopathy and social factors, for instance, racial disparities in peripartum cardiomyopathy (PPCM) or the social setting of hypertrophic cardiomyopathy. However, there have been insufficient materials to consider the relationship between social factors and clinical course in non-ischemic cardiomyopathies. There were various methodologies in therapeutic interventions, such as pharmacological, surgical, or rehabilitational, and educational issues. However, interventions that could be closely associated with social inequality have not been sufficiently elucidated. We will summarize the effects of social equality, which could have a large impact on the development and progression of HF in non-ischemic cardiomyopathies.

## Introduction

**Figure 1:**
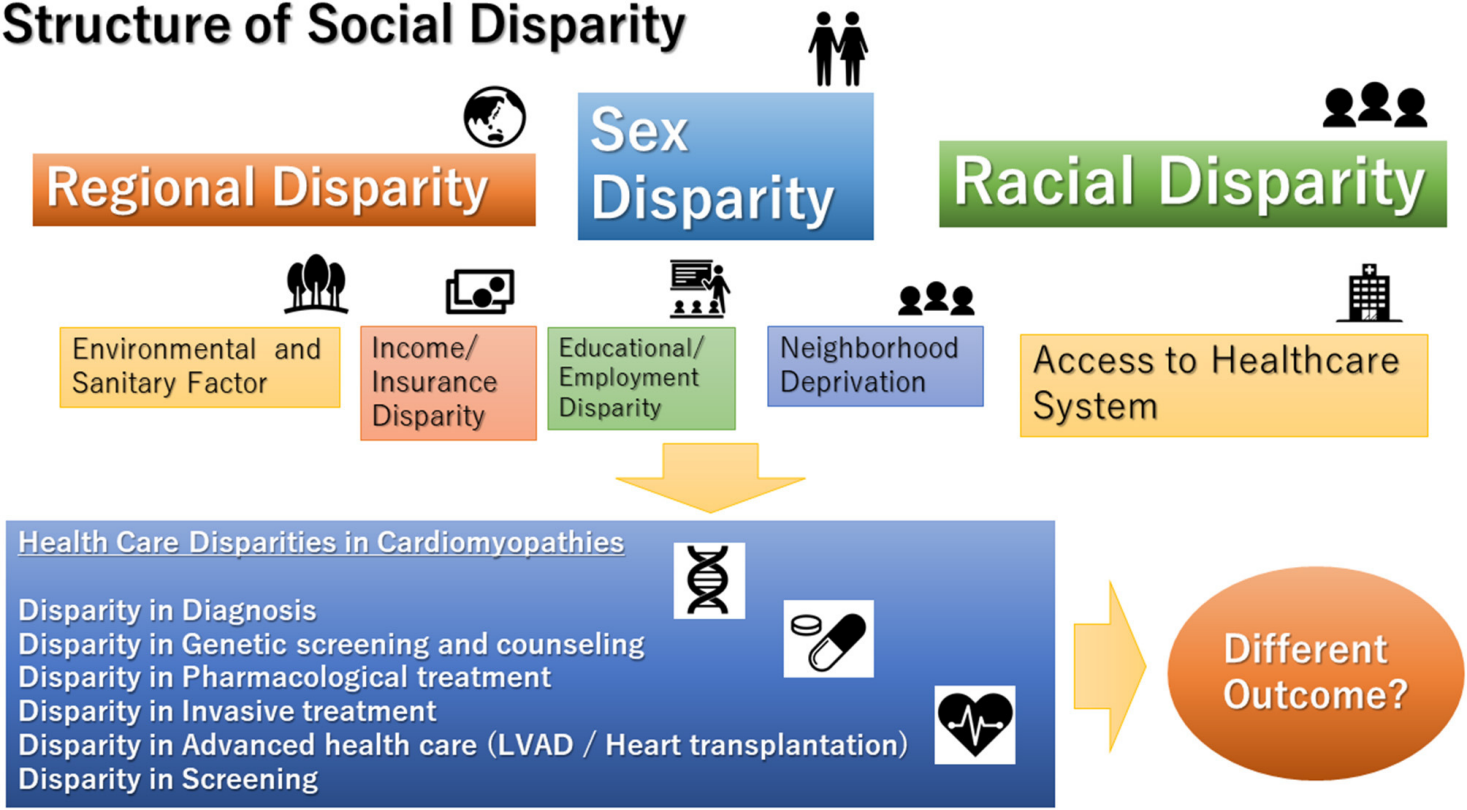
Structure of social disparities in cardiomyopathies. LVAD, left ventricular assist device.

The disparity is a topic that has recently attracted attention again in the health service. There have been increasing numbers of reports on the disparities in clinical interventions in the field of heart failure (HF) (1–3). In particular, women and racial minorities are likely to be subjected to inequities in medical therapy (4, 5). In fact, the clinical course of HF is significantly affected by social and environmental factors (2). In non-ischemic cardiomyopathy, there are some studies on the association between social parameters and clinical course. However, the relationship between the social environment and the clinical course of the disease varies from place to place, and it is difficult to create a universal review. In particular, the analysis of regional disparities across nations is extremely complicated. Due to differences in economic conditions, medical system, and culture, it is difficult to analyze the disparity between nations (6). To make the issue more solvable, one valid method is to analyze the effect of social factors in the same background of culture and medical system. As mentioned above, efforts to make it universal are necessary. Furthermore, it should not be overlooked that individual clinical studies may also have implications for social disparities (Figure 1).

Heart failure itself is a group of diseases closely related to the social environment, but cardiomyopathy, which often has a genetic background, may have a different relationship with the social environment. In this review, we focus on the different aspects of this relationship.

A recent publication analyzed the association between economic factors and the clinical course of HF for each Gini tertile, a summary measure of income or wealth inequality, in different parts of the world over different countries (7). It demonstrated that patients from lower-income countries [hazard ratio (*HR*) 1·58, (95% *CI* 1·41–1·77)], or with greater income inequality [from the highest Gini tertile; 1·25, (1·13–1·38)] had a higher 1-year mortality compared with patients from regions with higher income or lower income inequality. The relationship with the socioeconomic factor differs depending on the cause of HF. Acute HF is associated with high postdischarge mortality, particularly in patients with HF with reduced ejection fraction from low-income regions with high-income inequality, while patients with HF with preserved ejection had lower 1-year mortality with little variation by income level. From the report of Asia, there was significant heterogeneity among Asian patients with stable HF and the important influence of ethnicity and the level of regional income on the characteristics of the patients (8).

In particular, non-ischemic cardiomyopathy has the following special aspects. Many have a family history, often having a genetic abnormality as a factor; many cases have a long-term disease, such as juvenile-onset; and there is a certain frequency of high-severity cases due to the long-term disease. It can have a strong influence on life courses, such as pregnancy and employment (9). Furthermore, because the frequency of the disease is comparatively low, medical care from a professional point of view may be superior.

In fact, the rate of premature morbidity and mortality remains unacceptably high in cardiomyopathy (10); therefore, there may be an element in which the disparity is likely to become apparent (11). Furthermore, socioeconomic status (SES) might affect the adherence of the patient to medical advice and therapies (12). In addition, low income was associated with a higher mortality rate with lower ambulatory-based healthcare resources (13).

Generally, there were common pathways of disparities, such as hospital bed density, health worker density, education index, and race. Regarding the difference in a clinical course due to the difference in race, there may be a genetic factor, but there is a possibility that it is a result that reflects the difference in the social environment depending on the race. Differences in the region of residence might also affect disparities in clinical management. Pierce et al. (14) reported different trends in HF-related mortality between rural and urban regions in the United States. Age-adjusted mortality rates were consistently higher for residents in rural compared with urban counties [73.2 (95% *CI*: 72.2–74.2) vs. 57.2 (56.8–57.6), respectively]. Residence in socioeconomically disadvantaged communities also affected clinical courses (15). In addition, the disparity derived from a medical professional is possible, such as the shortage of cardiac professionals. In fact, the echocardiographic assessment is critically required to diagnose non-ischemic cardiomyopathy (16). A study in Denmark demonstrated that the diagnosis of cardiac dysfunction in the early stage assessed by echocardiography was associated with the educational level (17).

There were several disparities according to the treatment of HF. A previous study in Sweden reported reduced access to angiotensin-converting-enzyme inhibitor (ACEI) treatment in unemployed patients, which demonstrated the adjusted odds ratio (*OR*) for no ACEI dispensation for unemployed patients of 1.59 [95% *CI* 1.46–1.73] (18). It showed that low employment status, low-income level, low educational level, or birth in a foreign country affected the continuation of ACEI. Meanwhile, the study in the United States did not demonstrate racial differences in the survival benefit derived from the use of beta-blockers (19). According to the up-titration of beta blocker to the optimal dose, some disparities might be developed by the availabilities of the support for good adherence (20, 21). In recent trials that evaluated the effects of sacubitril/valsartan in the United States, no particular differences in effects were observed depending on race (22). In contrast, a study published in Denmark showed that attendance in cardiac rehabilitation is affected by social factors, such as educational attainment (23). The study in Denmark demonstrated inequalities in the care of HF by educational or income level (24). For instance, an income in the lowest tertile was associated with lower odds of prescription of ACEI/ARB [adjusted *OR* 0.80, (95% *CI*: 0.67–0.95)] and beta-blockers [adjusted *OR* 0.88, (95% *CI*: 0.86–1.01)], referral to exercise training [adjusted *OR* 0.59, (95% *CI*: 0.53–0.64)], and patient education [adjusted *OR* 0.66; (95% *CI*: 0.59–0.74)] compared with an income in the highest tertile. Regarding device treatment, the rate of implantable cardioverter-defibrillator implantation is low in females (25). In terms of sudden death, low educational level and low neighborhood SES were independently associated with an increased incidence of sudden cardiac death (26). The reasons included different levels of psychological stress and access to primary care and emergency medical services. According to the cardiac resynchronization therapy (CRT), the U.S. registry showed that it suffered from utilization disparities: male patients received more CRT devices compared with female patients; and whites received more CRT devices compared with Blacks (27). In addition, a recently published report from the U.S. database demonstrated sex disparities in choice of CRT device, with women less likely to receive a CRT-defibrillator (D) device compared with CRT-(pacing) P. They also showed that the patients in urban hospitals or higher bed capacity were more likely to receive CRT-D (28). Moreover, insurance status affects the decision of implantation of CRT (29). As described above, the disparity may occur in various clinical aspects in HF.

## Hypertrophic Cardiomyopathy

Hypertrophic cardiomyopathy (HCM) is a common genetic heart condition affecting the myocardium. There is a wide range of variability in the clinical course of HCM; however, the most important clinical events are the development of HF, atrial fibrillation, and sudden death (30).

The clinical course of HCM is determined by several factors, such as genetic status, echocardiographic findings, and pathological features. Patients with HCM are recommended to obtain shared decision medical care (31). Team-based specialized care is optimal for the improvement of patients with HCM.

According to the impact of socioeconomic factors, there was a fine review about racial disparity in HCM (32). In addition, Thomas et al. performed a retrospective cohort study of HCM that investigated the clinical course and SES and specialty care in the Yale inherited cardiomyopathy program. They showed that socioeconomically vulnerable patients with HCM had a higher mortality rate when they were not referred to specialty care (33). Low SES patients without the specialty care cohort had significantly higher all-cause mortality compared with high SES patients [adjusted *HR* 10.06 (95% *CI* 4.38–23.09; *p* < 0.001)]. On the contrary, no significant differences were observed in the clinical course between different SES if patients were referred to a specialized HCM care team. Ingles et al. (34) demonstrated that SES determined health service use or clinical outcomes in the HCM setting. There was an overrepresentation of patients from very advantaged and major metropolitan areas, suggesting that the inaccessibility of a specialty clinic was of great importance for the clinical course. In fact, team-based care was provided by cardiologists, electrophysiologists, surgeons, genetic counselors, medical engineers, transplant coordinators, and social workers. Patients with HCM should be regularly evaluated with multifaceted modalities, such as electrocardiographic monitoring, echocardiography, MRI, and cardiopulmonary exercise tests. This point also suggests the superiority of team-based management.

Recently, there was a large-scale study on the relationship between race and clinical outcome of HCM, including genetic abnormalities using a U.S.-based cohort. The study demonstrated that Black patients were less likely to be referred for subspecialty HCM care, less likely to undergo invasive septal reduction (14.6 vs. 23.0%; *p* = 0.007), and less frequently underwent genetic tests (26.1 vs. 40.5%; *p* = 0.006) (35). Furthermore, Black patients had a higher percentage of advanced HF with NYHA III and IV. Race-derived disparities consist of inequalities in care provision, decreased recognition of the disease, delays in timely management, barriers to accessing care, and environmental factors, such as lower SES. This factor seems to apply to other cardiomyopathies.

Additionally, there are disparities in the detection of genetic abnormalities. Interestingly, there may not be sufficient variant classification algorithms for patients in minority group populations because robust genomic investigations are not performed using reference cohorts in the genotyping of minority patients (36).

In terms of treatment, implantable cardioverter-defibrillator devices (ICD) are underused in women and racial minorities regardless of demographics, hospital characteristics, and comorbidities (37).

## Dilated Cardiomyopathy

Dilated cardiomyopathy (DCM) is a common cause of HF among various cardiomyopathies (38). DCM is familial in 20–50% of cases, and various exposures to an additional insult, such as chemotherapy, might determine the development of HF. On the contrary, the sanitary environment might affect the development of DCM because there were several findings on the association between viral infection and DCM. Miura et al. demonstrated that the lower education levels and cold or hot workplaces exhibited a significant association with the development of DCM (39). Some heavy metals were associated with DCM probability (40). Although there are still few reports on the relationship between environmental factors and DCM, it is one of the routes of disease disparity, such as the sanitary environment, and further research is required in the future. However, there may be some unknown interaction between the genome and environmental circumstances (41).

The difference by race was also demonstrated in DCM (42). Coughlin et al. (43) demonstrated that Blacks had an increased risk of developing idiopathic DCM with relative odds of 2.6 in the United States. They explained that the increased risk for Blacks was not due to income, educational attainment, alcohol consumption, cigarette smoking, or a history of hypertension, obesity, diabetes, or asthma. In contrast, Khan et al. demonstrated a higher all-cause mortality in Black patients with cardiomyopathy [*HR*: 1.15, (95% *CI* 1.07–1.25; *p* < 0.001)] (44) and demonstrated that it was possibly due to the inadequate delivery of treatment and access to care.

The diagnosis of DCM can be triggered by HF, but there are also some cases in which cardiac dysfunction is diagnosed by regular examination and cases in which cardiac dysfunction is confirmed by the diagnosis of related family members. Indeed, the addition of genetic tests in asymptomatic relatives of patients with DCM to guide periodic clinical surveillance is cost-effective (45, 46). However, the situation where such a system, e.g., genetic counseling, is actually possible has not been generally achieved, and in that sense, there is a disparity due to regional and socio-economical differences.

Based on the treatment, the current management for DCM does not vary from that of HF. However, as the cause of DCM is investigated in the future, more upstream treatment will be performed (47). Specifically, the ultimate therapy to address genetic abnormalities might improve the clinical course in patients with DCM with a genetic abnormality background. It is almost but not yet realized, but when it is realized, the aspect of professional treatment will become stronger and a disparity may occur.

More specialized medical care is required for secondary cardiomyopathy, and there will be a significant risk of diversity. However, it seems to be beyond the scope of this study.

In a U.S. cohort of pediatric cardiomyopathy, African-American and Hispanic children hospitalized with myocardial disease, such as myocarditis, exhibited approximately 30% higher odds for mortality than their Caucasian counterparts (48). The authors considered that barriers to care before hospitalization could contribute to disparate disease severity, leading to a different outcome.

## Peripartum Cardiomyopathy

Peripartum cardiomyopathy (PPCM) is a disease of systolic HF that occurs toward the end of pregnancy and months after delivery in the absence of preexisting heart disease. Recently, genetic predisposition in PPCM was reported to be shared with those in DCM (49). Therefore, the problem in PPCM has some similarities with that in DCM. However, there were some specific reports on PPCM. In a large cohort of women with PPCM in the University of Pennsylvania Health System, there were different clinical courses between various racial backgrounds (50). African-American women were diagnosed later in the postpartum period and were more likely to have a significantly reduced ejection fraction (<30%) (56.5 vs. 39.5%, *p* = 0.03), leading to higher recovery failure. When examining its mechanism, the same group demonstrated a neighborhood concentrated disadvantage index independently associated with adverse outcomes in women with PPCM (51). It is demonstrated that low area-based education persisted as significantly correlating with sustained cardiac dysfunction [relative risk (*RR*) 1.49; (95% *CI* 1.02–2.17)]. The report from Europe showed that the mode of presentation was largely similar, while there were marked differences in sociodemographic parameters, such as the Human Development Index and Gini index of inequality in patients with PPCM from different regions (52). Many reports revealed that SES has a strong influence on outcomes in relation to pregnancy (53–55), and it is considered that there is an element that causes the impact of SES on PPCM as a result of the affinity between pregnancy and SES.

On the contrary, there were some studies on the environmental factors in the development of PPCM. There were non-racial regional disparities in the clinical characteristics and outcomes of patients with PPCM in Nigeria, which might partly be explained by selenium supplementation (56).

## Left Ventricular Assist Device

Advanced cases of HF in cardiomyopathy are managed using LVAD or heart transplantation. Significant differences in left ventricular assist device (LVAD) implantation were based on sociodemographic risk factors. However, it should be fully considered that this issue is highly dependent on the medical system.

Using the United Network for Organ Sharing (UNOS) database, Okoh et al. performed a retrospective cohort analysis of patients who were implanted with a continuous flow LVAD between 2008 and 2018. There was no difference in survival between the respective races (57). Conversely, Breathet et al. (58) suggested that the LVAD implantation rates for Blacks did not increase proportionally, suggesting continued racial disparities, possibly due to the underinsurance or lack of social support.

In contrast, the area deprivation index had little impact on survival after LVAD implantation (59). Another study showed that low SES might not affect the clinical course after LVAD implantation (60). The readmission ratio was also not changed by the low SES (61). SES does not independently impact the survival and readmission after HeartWare HVAD and Heartmate III LVAD implantation. These findings suggested that the clinical course after LVAD implantation is not significantly affected by social background.

## Heart Transplantation

Based on heart transplantation, there are wide-range differences in the efficacy and situation of organ donation between different places (62, 63). Therefore, in addition to the disparity due to the local and social environment, some factors depend on each socioeconomic state in each place, although it is greatly influenced by the situation of transplantation in each place. Based on this, socioeconomic disparities were reported in the UNOS registry. Low SES and low educational levels were associated with poorer outcomes after a heart transplant, resulting in approximately 20–30% increased risk (64). There were similar reports on heart transplantation in a wide range of populations (65, 66); one mechanism that explains its adherence to the treatment after heart transplantation, such as appropriate immunosuppressive medications. Furthermore, race is considered a critical factor that determines the clinical course after heart transplantation. According to the UNOS registry, after adjusting for recipient, transplant, and socioeconomic factors, Black recipients had a significantly higher risk of posttransplant mortality (67). Financial limitations might influence the adherence to follow-up visits in the posttransplant period. In patients who were successfully bridged with an LVAD to heart transplantation, similar reports demonstrated that the African-American race is associated with the increased rates of graft failure after transplantation and decreased in 5-year survival compared with the Caucasian race (68).

Recently, the SES disparities decreased over time (67). The gap between the middle and highest classes decreased; however, the lowest SES still exhibited a significant risk over time. Sex disparities also existed in pediatric heart transplantation (69). There were also clinical disparities in the decision-making process of clinicians to allocate advanced heart therapies, such as LVAD and heart transplantation, due to biased patient state recognition (70, 71).

## Limitation

There are several limitations to this study. First, the sex disparity could not be adequately considered in this study. Unlike race disparity, sex disparity may be more closely related to differences in biological background and social involvement, requiring a more careful delineation. However, the complicated issue should be analyzed in a more concise way in the future. Second, in this study, the social disparity in non-ischemic cardiomyopathy was often examined, inferred from the racial disparity, which was often derived from the U.S.-based research. Indeed, few reports of the disparity are due to regional differences, economic conditions, or social conditions. However, it is necessary to study the impact of actual social disparity in non-ischemic cardiomyopathies.

## Conclusion

There may be publication bias for the disparity. Many studies in this time of disparity reported racial differences, but this difference depends on the region. Although sufficient scores for factors, such as accessibility to specialized facilities, caregiver support, and community awareness, have not been evaluated adequately, the analysis on social disparity remains inadequate. As described above, various levels of disparity occur due to various factors in society, affecting the clinical course of the disease. Efforts to reduce disparities require not only summarization through manuscript publication, but also accurate information on understanding, administrative considerations, and countermeasures at each site. Although some factors are difficult to obtain for universal findings, this study actually leads to clinical results and should not be overlooked or considered a necessary study issue.

## Author Contributions

EA performed conceptualization, methodology, data curation, validation, original draft preparation, writing, reviewing, and editing.

## Conflict of Interest

EA belongs to the Department, endowed by NIPRO-Corp, Terumo-Corp., SenkoMedical-Instrument-Mfg., Century-Medical, Inc., ONO-pharmaceutical-Co., Ltd., Medtronic-JAPAN Co., Ltd, Nippon-Shinyaku Co., Ltd, Abiomed-Inc, AQuA-Inc, Fukuda-Denshi Co., Ltd, Mochida-Pharmaceutical-Co.; Boehringer-IngelheimPharmaceuticals Inc., and Sun-Medical-Technology-Research Corp.

## Publisher's Note

All claims expressed in this article are solely those of the authors and do not necessarily represent those of their affiliated organizations, or those of the publisher, the editors and the reviewers. Any product that may be evaluated in this article, or claim that may be made by its manufacturer, is not guaranteed or endorsed by the publisher.
